# Supplementary material for: Lifelong Machine Learning Potentials for Chemical Reaction Network Explorations
Source: J Chem Theory Comput. 2025 Sep 22;21(19):9641–56. doi: 10.1021/acs.jctc.5c01127 (PMC12529901; doi:10.1021/acs.jctc.5c01127)
Supplement: Supplementary file 1 [file ct5c01127_si_001.pdf]

SUPPORTING INFORMATION:
Lifelong Machine Learning Potentials for Chemical Reaction Network Explorations

Marco Eckhoff* and Markus Reiher*

ETH Zurich, Department of Chemistry and Applied Biosciences, Vladimir-Prelog-Weg 2, 8093 Zurich, Switzerland.

E-mail: eckhoffm@ethz.ch; mreiher@ethz.ch

S1 Supporting Methodology

S1.1 High-Dimensional Neural Network Potentials

In a second-generation high-dimensional neural network potential (HDNNP),[1–3] a system's energy is calculated as a sum of atomic energy contributions $E^m_{\mathrm{atom},n}$,

$$E = \sum_{m=1}^{N_\mathrm{elem}} \sum_{n=1}^{N^m_\mathrm{atom}} E^m_{\mathrm{atom},n} \ . \tag{1}$$

The atomic energy contribution for each of the N_elem chemical elements and N^m_atom atoms n of element m, is represented by a feed-forward neural network,

$$E^m_{\mathrm{atom},n} = b^{m,3}_1 + \sum_{\lambda=1}^{n_2} a^{m,23}_{\lambda 1} \cdot f^2 \left\{ b^{m,2}_\lambda + \sum_{\kappa=1}^{n_1} a^{m,12}_{\kappa\lambda} \cdot f^1 \left[b^{m,1}_\kappa + \sum_{i=1}^{n_G} a^{m,01}_{i\kappa} \cdot \alpha^m_i \left(G^m_{n,i} - \beta^m_i \right) \right] \right\} \ . \tag{2}$$

HDNNPs typically apply non-linear activation functions $f(x)$ such as the scaled hyperbolic tangent.[4] The number of neurons in the hidden layers, n_1 and n_2, as well as the number of hidden layers itself, which equals two in Equation (2), are hyperparameters of the neural network architecture. They can be adjusted manually or by a neural architecture search method like Network Expressivity by Activation Rank (NEAR).[5] The input of the neural network represents the local atomic environment of atom n in form of a vector $\mathbf{G}^m_n$ with dimension n_G. The weight parameters a, b, α, and β are the same for atoms of the same element m. We

note that α and β are utilized to standardize the input values.[4] The weight parameters are optimized based on target energies and atomic forces of a training set of chemical structures. Atomic forces are obtained as negative energy gradients with respect to the atomic positions.

S1.2 Element-Embracing Atom-Centered Symmetry Functions

In the input vector, we employ element-embracing atom-centered symmetry functions (eeACSFs).[4] They are many-body representations utilizing interatomic distances and angles within a cutoff sphere. In addition, they take into account element information to represent multiple elements without the need for additional vector entries for each element combination. The eeACSF vector size is independent of the atomic environment, which is required for the application in pre-trained feed-forward neural networks. As eeACSFs do not exploit connectivities, they are able to describe chemical reactions. Moreover, translational, rotational, and permutational invariances of the potential energy surface are fullfilled.

In general, eeACSFs are based on products of a structural term F and an element-dependent term H. These products are summed and a scaling function S can be applied subsequently. Radial eeACSFs,

$$G_{n,i}^{\mathrm{rad}} = S\left[\sum_{j\neq n}^{N_{\mathrm{atom}}} H_{i,j}^{\mathrm{rad}} \cdot F_{i,j}^{\mathrm{rad}}\left(R_{nj}\right)\right] , \tag{3}$$

employ only interatomic distances R_{nj} between the central atom n and the neighbor atom j, while angular eeACSFs,

$$G_{n,i}^{\mathrm{ang}} = S\left[\sum_{j\neq n}^{N_{\mathrm{atom}}} \sum_{k<j\wedge k\neq n}^{N_{\mathrm{atom}}} H_{i,jk}^{\mathrm{ang}} \cdot F_{i,jk}^{\mathrm{ang}}\left(\theta_{njk}\right) \cdot F_{i,j}^{\mathrm{rad}}\left(R_{nj}\right) \cdot F_{i,k}^{\mathrm{rad}}\left(R_{nk}\right)\right] , \tag{4}$$

include also interatomic angles θ_{njk} between atom n and the neighbor atoms j and k.

For the representation of the radial structure, we propose in this work the function

$$F_{i,j}^{\mathrm{rad}}\left(R_{nj}\right) = \begin{cases} \exp\left[\eta_i - \eta_i \left(1 - \dfrac{R_{nj}^2}{R_{\mathrm{c}}^2}\right)^{-1}\right] & \text{for } R_{nj} < R_{\mathrm{c}} \\ 0 & \text{otherwise} \end{cases} , \tag{5}$$

with $\eta_i > 0$. This function does not require an additional cutoff function as applied in most previous works,[4,6,7] since it damps the value and all derivatives of the eeACSF smoothly to zero at the cutoff radius

R_c. For the angular structure representation, we introduce

$$F^{\mathrm{ang}}_{i,jk}\left(\theta_{njk}\right) = \exp\left\{\xi_i - \xi_i\left[1 - \left(\lambda_i - \frac{\theta_{njk}}{\pi}\right)^2\right]^{-1}\right\} \ , \tag{6}$$

with $\lambda_i = 0, 1$ and $\xi_i > 0$, as an alternative to the conventional cosine function.[4,6,7] A set of eeACSFs i applying different parameter values for η_i, λ_i, and ξ_i yields the structural fingerprint vector of the local atomic environment of atom n.

To resolve a structure's elemental configuration, the fingerprint vector contains element-dependent terms H with different element properties h that exploit the trends of the periodic table. These properties are the element's period number n, the group number in the s- and p-block m, i.e., main group 1 to 8, and the group number in the d-block d,

$$h_{i,j} \in \left\{1,\ n_j,\ m_j,\ d_j,\ \overline{n}_j,\ \overline{m}_j,\ \overline{d}_j\right\} \ . \tag{7}$$

For a balanced representation of light and heavy elements and those with few and many valence electrons, the reverse counting is provided by $\overline{n}_j := X - n_j$, $\overline{m}_j := 9 - m_j$, and $\overline{d}_j := 11 - d_j$. For main group elements, $d = \overline{d} = 0$ is applied. To obtain element-independent eeACSF, $h_{i,j}$ can be set to 1. The f-block can be included analogously to the d-block. We note that a single d- or f-element can often be sufficiently represented by setting $m = 2$ and $\overline{m} = 7$ for this element. In this work, we employ $X - 1 = 5$ as maximum period number, excluding elements heavier than xenon.

In the radial element-dependent terms, the element properties of the neighbor atoms j are divided by their respective maximal possible value $h_i^{\max}$ of the element property h employed in eeACSF i,

$$H^{\mathrm{rad}}_{i,j} = \frac{h_{i,j}}{h_i^{\max}} \ . \tag{8}$$

In this way, the contribution of each neighbor atom j is between 0 and 1. For the angular element-dependent terms, linear combinations, with hyperparameter $\gamma_i = \pm 1$, are applied for the element properties of the neighbor atoms j and k,

$$H^{\mathrm{ang}}_{i,jk} = \frac{\left|h_{i,j} + \gamma_i h_{i,k}\right| + \frac{1-\gamma_i}{2} C_{ijk}}{h_i^{\max}\left(\frac{1+\gamma_i}{2} + 1\right)} \ , \tag{9}$$

with

$$C_{ijk} = \begin{cases} 0 & \text{for } h_{i,j} = h_{i,k} = 0 \\ 1 & \text{otherwise} \end{cases} . \tag{10}$$

To ensure that the contributions are non-zero, the absolute linear combination is shifted by one for $\gamma_i = -1$. Exceptions are contributions for which both element properties are zero. For balancing the eeACSF values of different element properties, a division by $2h_i^{\max}$ for $\gamma_i = 1$ and by $h_i^{\max}$ for $\gamma_i = -1$ is applied.

To mitigate the strong dependence of the eeACSF value on the number of neighbors, a scaling function S can be applied. This approach can be beneficial when very different molecule sizes or particle densities are present, because otherwise the range of the eeACSF values can get very broad leading to a drop of parametrization performance. We propose the cube root-scaled-shifted (crss) scaling function,

$$S\left(G_{n,i}\right) = 3\left[\left(G_{n,i}+1\right)^{\frac{1}{3}} - 1\right] . \tag{11}$$

An alternative is, for example, a square root function $S(G_{n,i}) = G_{n,i}^{\frac{1}{2}}$.[4] Conventional atom-centered symmetry functions apply no scaling function,[6] equivalent to $S(G_{n,i}) = G_{n,i}$.

S2 Supporting Computational Details

S2.1 Element-Embracing Atom-Centered Symmetry Function Parameters

The cutoff radius of all eeACSFs is $R_\mathrm{c} = 6$ Å.

Table S1: Radial eeACSF parameters. All parameter combinations within a row are applied.

h	η
1	4.304151, 7.525644, 13.736394, 28.214130, 76.311166
m	5.179115, 9.125810, 17.147532, 37.635716, 120.860154
$\overline{m}$	3.571065, 6.235079, 11.143161, 21.760512, 52.169068

S2.2 Reference Element Energies

The reference element energies $E_\mathrm{elem}^\mathrm{ref}$ are calculated from a least squares fit of the total energies of H_2, CH_4, NH_3, and H_2O as a function of their stoichiometries.

Table S2: Angular eeACSF parameters. All parameter combinations within a row are applied.

h	γ	λ	ξ	η
1	1	0, 1	0.691206, 1.754422, 4.753861, 20.397713	6.053691, 12.572841, 34.260378
m	1	0, 1	1.012797, 2.554460, 7.748467, 55.451774	7.954147, 17.899930, 60.430077
m	−1	0, 1	0.839429, 2.112156, 6.007123, 31.736160	6.921566, 14.911981, 44.669257
$\overline{m}$	1	0, 1	0.452668, 1.217336, 3.110253, 10.268625	4.687023, 9.195906, 21.800865
$\overline{m}$	−1	0, 1	0.563491, 1.460994, 3.821960, 14.112051	5.317266, 10.707409, 27.028475

Table S3: The reference element energies $E_{\mathrm{elem}}^{\mathrm{ref}}$ in Hartree (E_{h}) based on PBE/def2-TZVP energies minus GFN2-xTB energies.

Element	$E_{\mathrm{elem}}^{\mathrm{ref}}$ / E_{h}
H	−0.09183154
C	−35.92108451
N	−51.80670081
O	−71.12306328

Table S4: The reference element energies $E_{\mathrm{elem}}^{\mathrm{ref}}$ based on PBE/def2-TZVP energies.

Element	$E_{\mathrm{elem}}^{\mathrm{ref}}$ / E_{h}
H	−0.58301215
C	−38.13106617
N	−54.75915835
O	−75.21088447

S3 Supporting Results

S3.1 Chemical Reaction Network

Table S5: Number of structures for different structure labels in the chemical reaction network benchmark data set. The structure label classifies the subtask in the *elementary step* trial from which the structure originates.

Structure label	PBE
complex optimized	4 629
elementary step optimized	119 926
minimum guess	28 014
minimum optimized	989
reactive complex scanned	64 809
TS guess	2 291
TS optimized	4 935
user optimized	2

S3.2 Lifelong Adaptive Data Selection

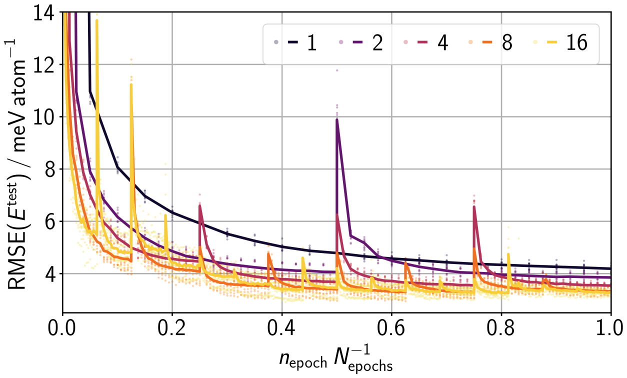

(a)

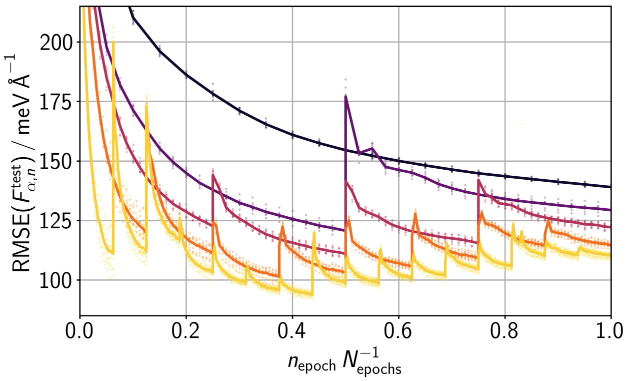

(b)

Figure S1: Test RMSEs of **(a)** energies E^{test} and **(b)** atomic force components $F_{\alpha,n}^{\text{test}}$ for training the data in 1, 2, 4, 8, and 16 sets. Each set was trained for 1 000 epochs, i.e., the total number of epochs N_{epochs} is higher for more sets. However, we note that a previously trained lMLP is available at no extra cost in many practical applications. The number of fitted structures per epoch was adjusted based on a constant fraction of the training structures not sorted out. $n_{\text{epoch}}\ N_{\text{epochs}}^{-1}$ represents a relative scale for the learning curves on the test data. The dots represent RMSEs of individual HDNNP ensemble members and lines show their mean.

S3.3 Lifelong Machine Learning Potentials for Exploration of Chemical Reaction Networks

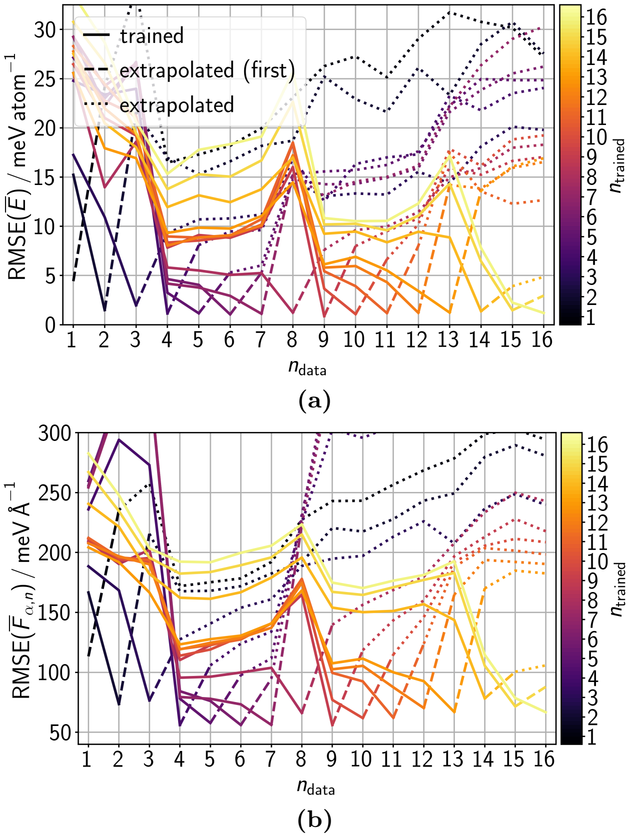

Figure S2: RMSEs of **(a)** energies $\overline{E}$ and **(b)** atomic force components $\overline{F}_{\alpha,n}$ evaluated with MLP ensembles at different training stages. In contrast to Figure 8 of the manuscript, lifelong learning was not applied, i.e., no rehearsal of training data (only training on newly added data), no lifelong adaptive data selection, and no stability-plasticity balance of the CoRe optimizer. The total CRN benchmark data was split into 16 chronologically ordered sets of equal size, with indices $n_{\mathrm{data}} = \{1, ..., 16\}$. The MLP ensembles were trained on the sets $n_{\mathrm{data}} = 1$ to n_{trained}. Each set was trained for 1000 epochs, whereby 750 randomly chosen structures of the training data set were considered per epoch. The training data set of each MLP ensemble member was a random subset of the respective sets. The RMSE evaluation of the MLP ensembles was based on all data of the respective set n_{data} and hence contained trained data. Lines are solid up to the last trained data set of the respective MLP ensemble. The extrapolation to the data set to be trained next is shown as dashed line, while further extrapolations are connected by dotted lines. Lines are shown to guide the eye, but only values at integer numbers of n_{data} are meaningful.

References

(1) Behler, J.; Parrinello, M. Generalized Neural-Network Representation of High-Dimensional Potential-Energy Surfaces. *Phys. Rev. Lett.* **2007**, *98*, 146401.

(2) Behler, J. First Principles Neural Network Potentials for Reactive Simulations of Large Molecular and Condensed Systems. *Angew. Chem. Int. Ed.* **2017**, *56*, 12828–12840.

(3) Behler, J. Four Generations of High-Dimensional Neural Network Potentials. *Chem. Rev.* **2021**, *121*, 10037–10072.

(4) Eckhoff, M.; Reiher, M. Lifelong Machine Learning Potentials. *J. Chem. Theory Comput.* **2023**, *19*, 3509–3525.

(5) Husistein, R. T.; Reiher, M.; Eckhoff, M. NEAR: A Training-Free Pre-Estimator of Machine Learning Model Performance. *arXiv:2408.08776 [cs.LG]* **2024**,

(6) Behler, J. Atom-centered symmetry functions for constructing high-dimensional neural network potentials. *J. Chem. Phys.* **2011**, *134*, 074106.

(7) Gastegger, M.; Schwiedrzik, L.; Bittermann, M.; Berzsenyi, F.; Marquetand, P. wACSF–Weighted atom-centered symmetry functions as descriptors in machine learning potentials. *J. Chem. Phys.* **2018**, *148*, 241709.
